# Importance of genetic sequencing studies in managing chronic neonatal diarrhea: a case report of a novel variant in the glucose–galactose transporter SLC5A1

**DOI:** 10.3389/fped.2024.1284671

**Published:** 2024-02-19

**Authors:** Lizbeth López-Mejía, Sara Guillén-Lopez, Marcela Vela-Amieva, Rosalía Santillán-Martínez, Melania Abreu, María Dolores González-Herrra, Rubicel Díaz-Martínez, Juan Gaspar Reyes-Magaña

**Affiliations:** ^1^ Laboratorio de Errores Innatos del Metabolismo y Tamiz, Instituto Nacional de Pediatría, Mexico City, Mexico; ^2^ Laboratorio de Biología Molecular, Genos Medica, Mexico City, Mexico; ^3^ Centro de Cáncer, Centro Médico ABC, Mexico City, Mexico; ^4^ Servicio de Medicina Interna, Hospital del Niño Dr. Rodolfo Nieto Padrón, Villahermosa, Mexico; ^5^ Servicio de Genetica, Hospital del Niño Dr. Rodolfo Nieto Padrón, Villahermosa, Mexico

**Keywords:** glucose–galactose malabsorption, infantile diarrhea, *SLC5A1*, SGLT-1, sodium/glucose cotransporter, inborn errors of metabolism, case report

## Abstract

**Introduction:**

Congenital glucose–galactose malabsorption (CGGM) is a rare autosomal recessive disorder that primarily causes chronic intractable diarrhea. This study aims to describe the clinical history, laboratory profile, diagnostic workflow, and management of the first patient reported with CGGM in Mexico.

**Methods:**

The case involves a Mexican female infant with recurrent admissions to the emergency room since birth due to chronic diarrhea.

**Results:**

The infant was born at term by C-section with a birth weight of 3.120 kg and height of 48 cm for consanguineous parents. She had been breastfed until day 5 of her life when she presented lethargy, diarrhea, abdominal discomfort, and jaundice. During the first evaluation at the emergency room, the significant laboratory finding was blood tyrosine elevation; afterward, amino acid and succinylacetone determinations were obtained, discarding tyrosinemia. When admitted to the hospital, an abdominal ultrasound detected a duplex collecting system. At this time, rice formula was introduced to the patient. She was discharged with jaundice improvement, but diarrhea persisted. Several formula changes had been made from rice to extensively hydrolyzed casein protein to whey-based, with no clinical improvement; the patient still had 10–12 excretions daily. In the second hospitalization, the patient presented anemia, severe dehydration, hyperammonemia, and renal tubular acidosis. A next-generation sequencing panel for inborn errors of metabolism and congenital diarrhea was performed, identifying a homozygous variant in *SLC5A1* (c.1667T > C). The diagnosis of CGGM was made at 3 months of age. The infant was initially treated with a modular galactose–glucose-free formula with oil, fructose, casein, minerals, and vitamins until a commercial fructose-based formula was introduced. This led to a complete resolution of diarrhea and improved nutritional status.

**Discussion:**

Diagnosing CGGM is challenging for clinicians, and next-generation sequencing is a valuable tool for providing appropriate treatment. More detailed information on patients with this condition might lead to possible phenotype–genotype correlations. This case's primary clinical and biochemical findings were chronic diarrhea, anemia, jaundice, renal tubular acidosis, hyperammonemia, and initial hypertyrosinemia. Symptoms were resolved entirely with the fructose-based formula.

## 1 Introduction

Diarrhea in neonates is defined by a stool volume of more than 20 ml/kg/d and a chronic one that lasts more than 14 days. One of the causes of chronic non-bloody diarrhea could be explained by an osmotic process of unabsorbed or partially absorbed nutrients, leading to an increased intraluminal solute load. Carbohydrate malabsorption is the most frequent finding related to osmotic diarrhea (1). Monogenic intestinal epithelial disorders or congenital diarrheas and enteropathies are a heterogeneous group of diseases characterized by neonatal or infantile-onset diarrhea and malabsorption. They are classified as defects in epithelial transport, epithelial enzymes and metabolism, epithelial structure, trafficking, polarity, enteroendocrine function, and epithelial stem cell function. The sodium-dependent glucose transporters could be responsible for congenital diarrhea; different genes are associated with this dysfunction: *SLC26A3* (MIM 126650)*, SLC10A2* (MIM 601295)*, SLC5A1* (MIM 182380), *SLC7A7* (MIM 603593), *SLC39A4* (MIM 607059), *GUCY2C* (MIM 601330), *SLC9A3* (MIM 182307), and *SLC51B* (MIM 612085) (2).

Congenital glucose–galactose malabsorption (CGGM) is an autosomal recessive genetic disorder (CIE-10 74.3, CIE-11 5C61.0) caused by pathogenic variants in *SLC5A1* (OMIM #606824, https://www.omim.org/entry/606824) encoding for the sodium-dependent glucose transporter-1 (SGLT-1) (3). SGLT-1 is the primary transporter of monosaccharides in the small intestine and is responsible for moving glucose and galactose across the intestinal brush border using active transport (4). A defect in the SGLT-1 results in the non-absorption of glucose, galactose, and sodium, leading to clinical manifestations that begin in the neonatal period (5).

The most common symptoms include severe hyperosmotic diarrhea, dehydration, abdominal distension, vomiting, failure to thrive, and weight loss (6). Other complications reported are kidney injury, hypernatremia, hypercalcemia, nephrolithiasis, polyuria, and hematuria (7). Diagnosis should be suspected based on clinical symptoms and confirmed by genetic testing (5). Treatment consists of a long-term particular dietary therapy that avoids foods containing glucose and galactose (8). Older children may show improved glucose tolerance as they age (9). In this study, we report a homozygous likely pathogenic variant in *SLC5A1* and describe the clinical and biochemical characteristics and management of the first patient with CGGM reported in Mexico.

## 2 Case presentation

The proband is an 8-month-old female, a child of second pregnancy, for consanguineous parents from a small endogamic region in Tabasco, Medellin de Madero, a town of nine thousand inhabitants. She was the second offspring in the family, with a previous healthy sibling. The patient's mother had prenatal care with four ultrasounds and five doctor visits during her pregnancy. The proband was born by C-section due to oligohydramnios. At birth, a weight of 3.120 kg (Z-score: −0.25) and a height of 48 cm (Z-score: −0.62), weight for length 0.52, were reported, Apgar 9. She was exclusively breastfed with adequate suction for 5 days when she presented with abdominal distension and diarrhea that gradually increased to 10–12 liquid stools daily. At 1 month of age, she was admitted to the hospital for 8 days for jaundice, lethargy, and severe diarrhea. During her first evaluation at the emergency room, coprological and coproparasitoscopic methods were performed without finding any alterations in the reported samples, and fecal-reducing substances were reported negative. Viral infections were also discarded. Because of the hepatic alterations, antibodies IgM and IgG against CMV and EBV were also tested, with negative results. C-reactive protein, thyroid function test, and coagulation parameters showed no alterations. Severe dehydration was immediately corrected with IV fluids, so a trial of oral rehydration solution was not performed. A metabolic screening showed elevated blood concentrations of tyrosine (6.57 mg/dl, normal range <4.98 mg/dl) and alpha-fetoprotein (184 ng/ml, normal range: 0–20 ng/ml) but average blood concentrations of succinylacetone. Afterward, tyrosine 46 µmol/L (normal range 26–155 µmol/L) and succinylacetone were repeated and were in the normal ranges, ruling out the diagnosis of tyrosinemia type 1. Relevant biochemical studies during treatment are depicted in Table 1. When admitted to the hospital, an abdominal ultrasound revealed a duplex kidney collecting system and a clubfoot was diagnosed. At this time, breastfeeding was suspended, and she was given a partially hydrolyzed formula based on rice protein. However, jaundice improved over time; there was no change in the number of bowel movements, so the formula was changed to extensive casein hydrolyzed, and then she was discharged.

**Table 1:** Relevant biochemical studies during treatment of a CGGM patient.

Biochemical studies	Age			
	1 month	8 months	9 months	10 months
Glucose (mg/dl)	75	102	95	76
Urea (mg/dl)	39	28	17.3	
Blood nitrogen urea (mg/dl)	18.4	13	8.1	
Creatinine (mg/dl)	0.6	0.2	0.4	0.3
Uric acid (mg/dl)		3.1	4.8	
Sodium (mmol/L)	145	137		
Potassium (mmol/L)	3.5	5.4		
Chlorine (mmol/L)	118	106		
Calcium (mg/dl)	12.2	10.6		
Phosphorus (mg/dl)	5	7		
Magnesium (mg/dl)	2.1	1.9		
Triglycerides (mg/dl)	80	86	86	77
Total cholesterol (mg/dl)	138	142	134	210
HDL cholesterol (mg/dl)		67		74
LDL cholesterol (mg/dl)		57.8		113
VLDL cholesterol (mg/dl)		17.2		15.4
Total bilirubin (mg/dl)	11.2	0.3		
Direct bilirubin (mg/dl)	0.6	0.1		
Indirect bilirubin (mg/dl)	10.6	0.2		
Serum albumin (g/dl)	4.1	4.3		
Aspartate aminotransferase (U/L)	30.3	34		
Alanine transaminase (U/L)	24	26		
Hemoglobin (g/dl)	6.6	12.2	11	12.4
Hematocrit (%)	19.6	36.8	34	37.6
Platelet (×10^3^/μl)	641	541	545	367
pH	7.1			
HCO_3_ (mmol/L)	3.8			
Lactate (mmol/L)	2.5			
Ammonium (µmol/L)	213	29		

After several formula changes, the patient continued to have 10–15 excretions per day, causing her severe dehydration, vomiting, anorexia, and inadequate weight gain, requiring a second hospitalization at 2 months of age that lasted 80 days. During this hospitalization, another coprological study was done; this time, an acid stool pH of 5 and positive reducing substances were reported. The patient presented anemia (hemoglobin 9.6 g/dl, hematocrit 32.2%), hyperammonemia (213 µg/dl), hypernatremia with sodium levels initially at 145 mmol/L, increasing up to 152.8 mmol/L, and hypercalcemia of 8.9 mg/dl. The patient was diagnosed with severe malnutrition, and an ultrasound was performed with evidence of nephrocalcinosis. The nephrology department started sodium bicarbonate at 7 mEq/kg/d due to hyperchloremic metabolic acidosis, elevated urinary pH, and hypokalemia. Parenteral nutrition with IV glucose/kg/minute of 6.5 was initiated through a central line because the patient experienced hypoglycemia (69 mg/dl) even with enteral formula infusion through a nasogastric tube. The patient had two previous transfusions. The hematology department started erythropoietin at 2,000 IU per week and 1,000 IU twice weekly, along with iron, folic acid, and complex B vitamin supplementation to treat anemia. A positive blood culture for *Klebsiella pneumoniae* was also detected and treated with meropenem.

At this point, a monogenic disease was suspected due to the heterogeneous phenotype, and a next-generation sequencing panel was considered the first genetic test. Genomic DNA was extracted from peripheral blood using the Gentra Puregene Kit (Qiagen®). Clinical Exome Solution V3 Panel (Sophia Genetics, Saint-Sulpice, Switzerland) was used for in-solution hybridization to enrich target sequences, which covers the coding regions (±5 bp of intronic regions) of 4,728 genes, the entire mitochondrial genome, and ∼200 non-coding variants with known pathogenicity in deep introns/enhancer/promoter genes. The primers were by design (Primer3 v. 0.4.0) as follows: Chr22:32104691: Fw 5′-TTCCCTTCCTTGATGCATATCT-3′ Rv 5′-TTAACTTCCCCAAACCCACTT-3′. The DNA sample was sequenced on the Illumina NextSeq 500 (Illumina, Inc., San Diego, CA, United States). The average coverage depth was 113×, with over 99.8% of the target regions covered by at least 20 reads. Sequence quality control was performed with FastQC; BWA mapped reads, variant calling was done with GATK4, and verification of the effects of these variants using SnpEff, inspection of selected variants through IGV, and sequenced reads were compared with the reference human genome version (GRCh38/hg38).

The bioinformatic analysis was done in the Sophia DDM platform (Sophia Genetics, Saint-Sulpice, Switzerland) with a virtual gene panel of 1,125 genes associated with inborn errors of metabolism, 260 of congenital diarrhea and 100 with both phenotypes; 1,485 genes in total were analyzed (Supplementary Table S1).

**Figure 1:**
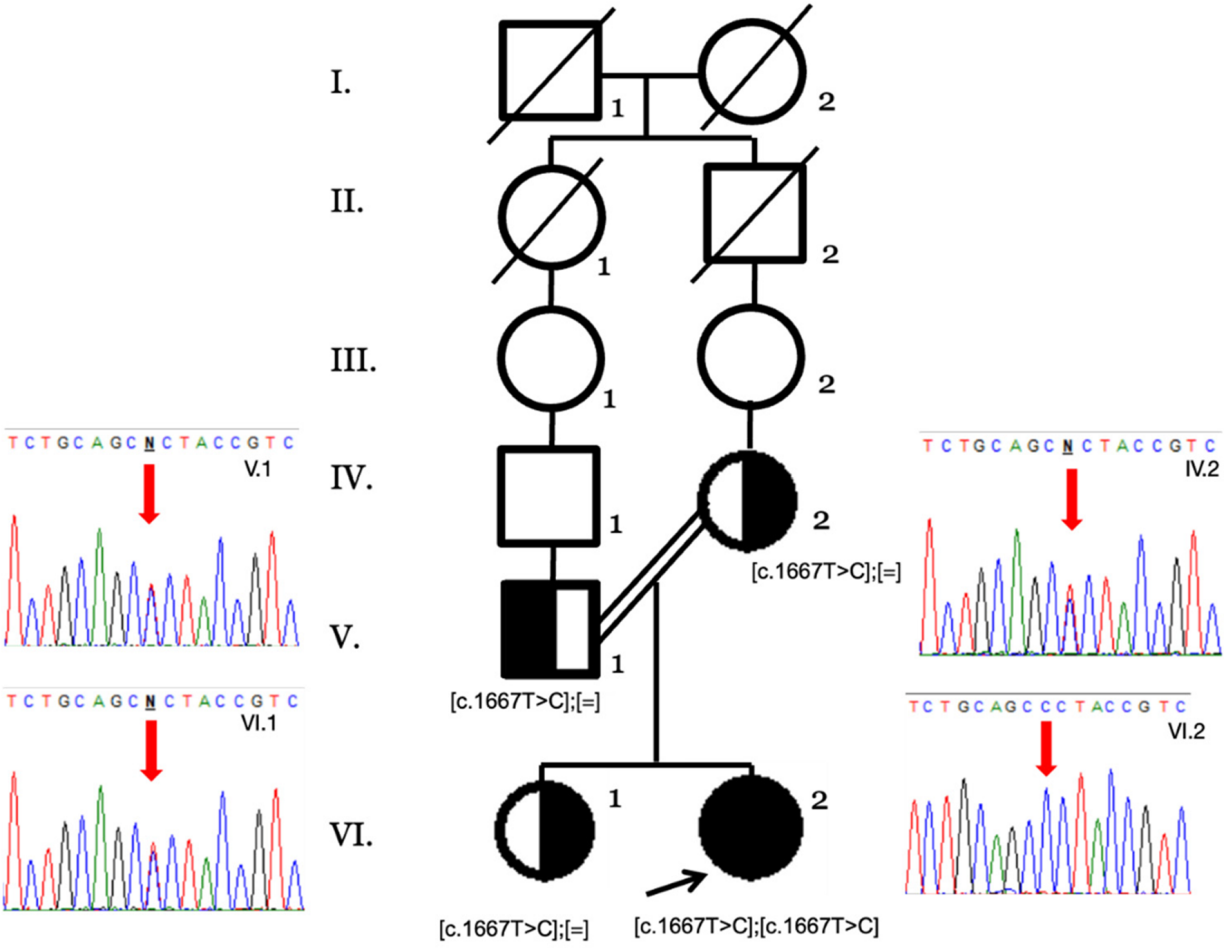
Family pedigree by Sanger sequencing of the available family members. The heterozygous genotypes of the nuclear family (V.1, IV.2, and VI.1) and the homozygous variant in the index case (VI.2) are shown with the red arrows.

This panel allowed the identification of the NM_000343.4 (SLC5A1):c.1667T>C (p.Leu556Pro) variant. This missense mutation replaces leucine residue by proline at position 556 of the coding region of the *SLC5A1* gene (exon 14 of 15) located in the single intramembrane position of the protein. It was classified as likely pathogenic according to the ACMG criteria (10) as it has extremely low frequencies in population databases and is not present in Clinvar, LOVD, or disease-related databases (PM2), for a missense variant in a gene that has a low rate of benign missense variation and in which missense variants are a common mechanism of disease (PP2). Several lines of computational evidence support a potential pathogenic effect (PP3, REVEL 0.875 score as pathogenic moderate, PolyPhen: possibly damaging, MetaRNN: pathogenic strong). The segregation analysis by Sanger sequencing confirmed the heterozygous state of both parents (PM3) (Figure 1), and the patient's phenotype history is highly specific for a disease with a single genetic etiology (PP4). CGGM was diagnosed at 3 months of age with the clinical phenotype and the likely pathogenic variant detected.

Specific dietary treatment for CGGM was started after diagnostic confirmation at the Pediatrics Hospital in Tabasco with the support of the National Pediatrics Institute in Mexico City. Since the fructose-based commercial formula (Galactomin 19® Nutricia, Germany) was not available in Mexico, a modular galactose–glucose-free formula containing oil, fructose, casein, vitamins, and minerals was introduced initially. When Galactomin 19® became available, it replaced the modular diet. Significant clinical improvement was observed, with a decrease in the number of bowel movements and improved nutritional status. The patient was discharged at 3 months of age. Over 2 months, from 4 to 6 months, the patient's mother chose to increase the consumption of fructose formula without following the prescribed guidelines. Consequently, the patient experienced a significant increase in weight and triglyceride levels (271 mg/dl). When the patient reached 6 months of age, medical follow-up was re-established and provided a specific prescription to reduce the amount of formula intake. Furthermore, we counseled the patient and her family about the potential adverse effects of consuming excessive fructose in her diet.

**Figure 2:**
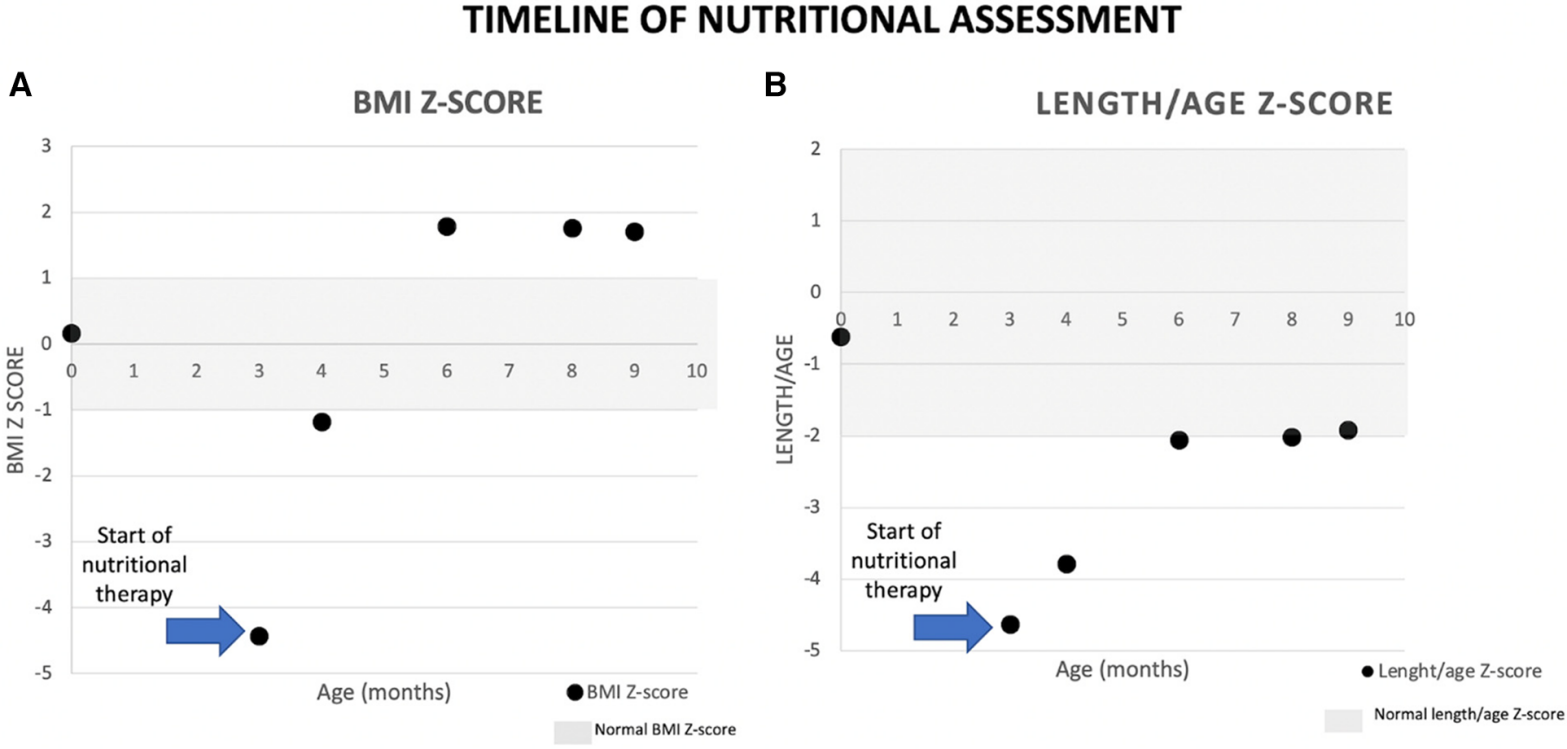
Patient´s anthropometric assessment.

Complementary feeding was started at 6 months of age, and a 1-month meal plan was given to the family to enhance adherence; the diet included fruit, vegetables, meat, and some small amounts of cereals. Currently, the patient presents one to two depositions per day, and constipation has also been reported but less frequent; she has a normal developmental milestone for her age. Renal function tests were normalized after the second hospitalization, and a citrate solution was prescribed until she was 6 months old. After that, the nephrologist prescribed hydrochlorothiazide 1.5 mg once a day to treat nephrocalcinosis. Nephrological follow-up will continue in Tabasco, and nutritional follow-up is ongoing at the National Institute of Pediatrics in Mexico City (Figure 2).

## 3 Discussion

**Figure 3:**
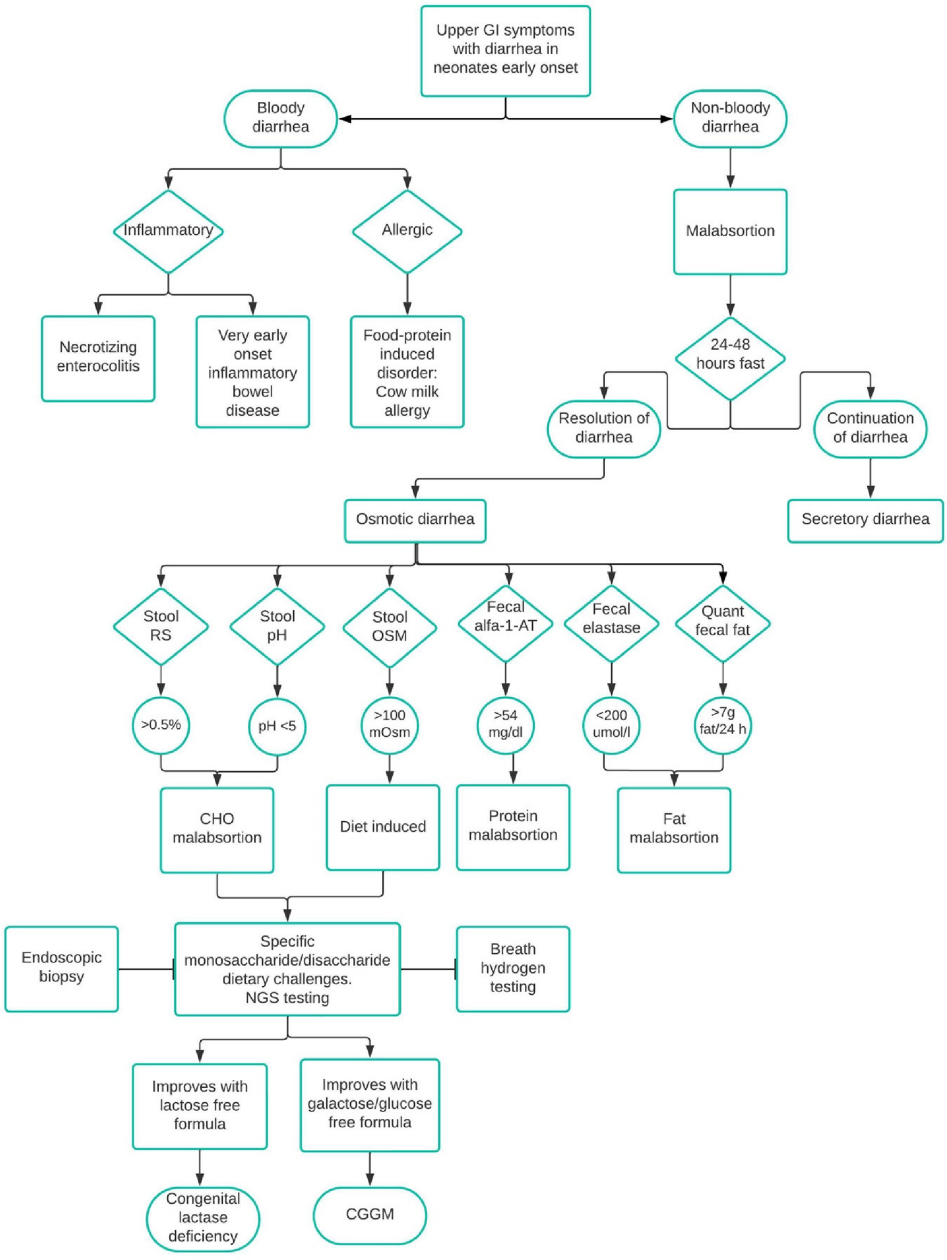
Differential diagnosis of diarrhea in neonates. CHO, carbohydrate; alfa-1-AT, alfa-1-antrypsin; OSM, osmolality; RS, reducing substances; Quant, quantitative; NGS, next-generation sequencing.

The diagnosis of CGGM can be challenging to suspect for clinicians because the symptoms are not specific (6). Reaching the correct diagnosis in neonates starts by discarding general causes of chronic diarrhea. A key point is the timing, mainly if it occurs soon after birth, because it could be related to congenital anomalies and enteropathies (1, 2). At the beginning of diagnosis, an important point to consider is to distinguish whether there is blood in the stools so inflammatory and allergic causes can be discarded. In this case, non-bloody diarrhea can be due to malabsorption. Medernach and Middleton suggest performing a 24–48 h fasting to differentiate between osmotic and secretory diarrhea. A stool pH < 5 suggests carbohydrate malabsorption because of its fermentation and the subsequent production of short-chain fatty acids. Stool-reducing substances greater than 0.5% is indicative of monosaccharide malabsorption. In the first hospitalization, the patient did not show a decreased pH in stools or positive reducing substances to suspect carbohydrate malabsorption, and it was until the second hospitalization that the patient manifested these alterations. Even though an esophagogastroduodenoscopy with biopsy could differentiate a deficiency among the disaccharidases, in this case, a biopsy cannot help establish the diagnosis of CGGM (11) nor the breath hydrogen testing. In some local hospitals, these procedures are not available. Figure 3 describes an algorithm for the differential diagnosis of diarrhea in neonates. As mentioned, several genes could be related to defects in epithelial transport, and some differences might lead to the diagnosis; for example, *GUCY2C*, *SLC9A3,* and *SLC26A3* are early-onset watery secretory diarrheas, unlike *SLC5A1* (2). However, establishing the diagnosis based on the clinical and biochemical findings might take a long time. Initial history and symptoms, serum laboratories, stool and dietary evaluation, endoscopic biopsy, and genetic testing should be performed (2, 11). In the patient reported in this case, the main symptom observed was severe osmotic diarrhea that began in the first days of life after feeding exclusively with breast milk. Because of the late treatment, she developed nephrolithiasis. In a recent literature review of CGGM, complications such as kidney injury, hypercalcemia, and nephrolithiasis were found in 18.7% of the studied patients, and hypernatremia (found in 53.3%) was also shown in our case (7).

Diarrhea led to severe dehydration, anemia, jaundice, renal tubular acidosis, hyperammonemia, and malnutrition. These symptoms are like those reported in the literature (6). Dietary treatment that consists of glucose and galactose elimination from the diet resulted in an essential clinical improvement and in the cessation of symptoms (8). The initial hand-crafted formula recipe is described in Supplementary Table S2. The diet should include a metabolic formula with fructose, fruits and vegetables, animal protein sources, legumes, oils, and fats. A more detailed list of allowed and restricted food is described in Table 2 (8, 12).

**Table 2:** Allowed and restricted food in congenital glucose–galactose transporter deficiency.

Group of food	Allowed	Restricted
Dairy products	None	All: milk, human milk, formulas with lactose, butter, yogurt, cheese, ice cream, industrialized food with lactose, condensed milk, cream, all sorts of dairy products
Fruits	All kinds: banana, apple, peach, strawberry, oranges, pear, watermelon, unsweetened juice	Fruits with added sugar, fruits in syrup, fruit juice with sucrose, marmalade, jellies
Cereals	Rice, noodles, potato, quinoa, wheat, unsweetened cereal, bread with fructose, oats, and whole grain without sugary coating	Sweetened cereal, biscuits, bread with sucrose and milk, all desserts made with sugar and milk, commercial cookies, and cakes
Vegetables	All kinds	None
Animal protein	All sorts: fish (salmon, tuna, catfish), egg, chicken, beef, turkey, duck, pork, lamb, goat, seafood (shrimp, crab, clams, lobster, scallops, oysters, mussels)	None
Legumes	Beans, soybeans, chickpeas, lentils, peas	None
Nuts	All: Cashews, peanuts, almonds, hazelnuts, walnuts, Brazil nuts, pecans, pine nuts, pistachios	Praline nuts with sugar
Fats and oils	All vegetable oils	Frostings, meringue with sugar
Sweeteners and flavorings	Fructose, honey, cocoa, sugar-free marmalade	Glucose, dextrose, dextrin, glucose polymers, maltodextrin, maltose, corn syrup, lactose, stevia, sugar (sucrose), candies, chocolate, ketchup with sugar

Early diagnosis is essential to improve symptoms and avoid complications such as kidney injury and nephrolithiasis. Health personnel must know about this metabolic disease to consider CGGM a possible diagnosis in newborns presenting severe osmotic refractory diarrhea, metabolic acidosis, and dehydration that do not respond to standard therapy (5).

As it has been previously described (13), patients with congenital diarrheal disorders benefit from genetic testing as the first diagnostic approach because they provide not only the specific diagnosis but also the rapid implementation of target therapies. In this case, a next-generation sequencing panel identified a homozygous missense variant in *SLC5A1* (c.1667T > C). *SLC5A1* (Gene/locus MIM number: 182380) is located on the long arm of chromosome 22 in the region 22q12.3. This gene encodes a member of the GLT family in epithelial cell membranes of the intestine that co-transports sodium ion(s) and monosaccharides into the cell using the sodium concentration gradient. *SLC5A1* is predominantly expressed in the small intestine, characterized by its strict selectivity and binding affinity to D-glucose (1.74 mM) and D-galactose (3.12 mM) substrates (14). This gene comprises 15 exons extending over 69.8 kb and encodes 664 amino acids. Fifty-six different missense, non-sense, frame shift, and splice site variants have been described in *SLC5A1* associated with CGGM; most are private coding variants that produce abnormal or truncated proteins. Family cases reported of CGGM with missense variants in *SLC5A1* have shown loss of the transporter activity that impairs trafficking to the plasma membrane (15). The variant c.1667T > C (p.Leu556Pro) affects the C-terminal part of the transporter (residues 407–664 containing trans-membrane helices 10–14), which determines sugar selectivity and affinity; this may explain the accumulation of unabsorbed glucose and galactose observed clinically in this patient.

In the first genetic assessment, the family was not self-aware of any consanguinity, only that they were coming from a small town (endogamy). Until the detection of the homozygous variant, a six-generation pedigree confirmed the relationship and genetic counseling as an autosomal recessive disorder was given to the family. Among 65.2%–76.7% of the CGGM cases reported are homozygous, such as this case, supporting the observation that this is a disease with high morbidity in countries or regions with high endogamy or inbreeding (6).

The variant c.1667T > C (rs1049516620) has an extremely low frequency reported in population databases (4/264,690, TOPMED; 3/140,234, GnomAD). One of the main disadvantages of contrasting low-frequency variants in public databases is that not all populations are well represented; however, in the internal records of the laboratory, which has more than 2,000 clinical exomes of Mexican patients and in the 79,656 clinical exomes from the Sophia Community, the variant was not detected either; this along with the segregation analysis suggest it as associated with the disease. There are only 107 documented CGGM cases from 2001 to 2019 worldwide (6). This rare variant probably has a founder effect in this Tabasco community, and the healthcare providers of the region must be aware of this metabolic disease presenting as chronic osmotic refractory diarrhea and treated promptly to avoid unnecessary prolonged hospitalizations, morbidity, and mortality.

It is essential to highlight the benefit of the next-generation sequencing panel performed on this patient, which allowed the correct diagnosis of a rare disease difficult to suspect clinically, especially in the neonatal period when presentation tends to be more severe with life-threatening episodes. Next-generation sequencing is now proposed also as a first-line test for diagnosing inborn errors of metabolism; this panel type has favorable diagnostic rates as high as 60% (16). Once the diagnosis was confirmed, the specific treatment saved this patient's life. Diagnosing an inborn error of metabolism is a medical challenge, particularly considering that CGGM does not appear in any newborn screening program, and this disease does not have a specific blood or urine biomarker.

A proper follow-up should remain throughout the duration of treatment. Excess fructose leads to obesity and hypertriglyceridemia, which can be preventable with a closer follow-up. Fructose promotes *de novo* hepatic lipogenesis with an overproduction of acetyl-CoA and glycerol-3-phosphate and is linked with postprandial hypertriglyceridemia, leading to hepatic steatosis, insulin resistance, and hyperuricemia (17). The biochemical follow-up in these patients should consider regular insulin, glucose, hemoglobin A1C, uric acid, lipid profile, and liver function test. Medical–nutritional assessments should prevail throughout the patient’s life to prevent complications.

In conclusion, clinicians should consider CGGM in patients with chronic diarrhea or renal tubular acidosis. Early diagnosis through next-generation sequencing approaches can save lives by initiating specific treatments promptly and avoiding a diagnostic odyssey. Regular follow-up and dietary management are essential to prevent complications and ensure better long-term outcomes for patients with CGGM. Healthcare professionals’ awareness of this metabolic disorder is critical to improving patient care.

## Data Availability

The datasets for this article are not publicly available due to concerns regarding participant/patient anonymity. Requests to access the datasets should be directed to the corresponding author.
