# Supplementary material for: Importance of genetic sequencing studies in managing chronic neonatal diarrhea: a case report of a novel variant in the glucose–galactose transporter SLC5A1
Source: Front Pediatr. 2024 Feb 19;12:1284671. doi: 10.3389/fped.2024.1284671 (PMC10909829; doi:10.3389/fped.2024.1284671)
Supplement: Supplementary file 2 [file Table2.docx]

Supplementary Table II. Initial hand-crafted formula recipe for a patient with CGGM

Patient´s weight 2.83 kg

| Food | Amounts | Carbohydrates | Protein | Fat |
| --- | --- | --- | --- | --- |
| Fructose powder | 35 g | 35 | -- | -- |
| Casein module (Casec™) | 10 g | -- | 8.8 | 0.1 |
| Vegetable oil with DHA (Nutrioli DHA ™) | 20 ml | -- | -- | 18 |
| Multivitamin without added sugar | 1/3 of tablet | -- | -- | -- |
| Add water to make | 480 ml (16 oz) |  |  |  |
| Total grams |  | 35 | 8.8 | 18 |
| Total calories | 337.2 | 140 | 35.2 | 162 |
| % |  | 41.5% | 10.4% | 48.2% |

169 ml/kg/d, 120 kcal/kg, 3.1 g/kg de protein, 21 kcal/per ounce
